# An In-House Method for Molecular Monitoring of BCR-ABL

**DOI:** 10.5152/tjh.2011.76

**Published:** 2012-12-05

**Authors:** Hakkı Ogün Sercan, Ilgın Öztürk, Ceyda Çalışkan, Melek Pehlivan, Zeynep Sercan

**Affiliations:** 1 Dokuz Eylül University, School of Medicine, Department of Medical Biology and Genetics, İzmir, Turkey

**Keywords:** Chronic myeloid leukemia, BCR-ABL, Real-time PCR, Molecular response

## Abstract

**Objective: **At present, there are a limited number of facilities in Turkey that can provide reliable real-time quantitative(RQ)-PCR BCR-ABL results. The present study aimed to test a cost-effective, in-house method of BCR-ABL quantification,including verification of the method by RQ-PCR validation tests.

**Material and Methods:** BCR-ABL and ABL target sequences were cloned into pJET1.2 vectors, from whichcalibrators were prepared and used as templates in RQ-PCR reactions to generate standard curves. Dilutions of K562cells (representing an in vitro simulation of BCR-ABL transcript reduction) were analyzed.

**Results:** Standard curves were generated from calibrators. These curves were then used to calculate the BCR-ABL andABL copy numbers; in which linear BCR-ABL results were obtained. Repetitive experiments showed that our methodologywas able to detect 1 BCR-ABL positive cell from amnong 1x105 cells.

**Conclusion:** The method described herein is suitable for implementation with any RQ-PCR instrument and/or kit forquantify BCR-ABL transcripts.

**Conflict of interest:**None declared.

## Abstract (tr)

**Amaç:** Türkiye’de BCR-ABL gerçek zamanlı kantitatif polimeraz zincir tepkimesi ile BCR-ABL düzey tayini yapabilen merkezlerin sayısı, ihtiyacın altında kalmaktadır. BCR-ABL kantitasyonu için laboratuarımızda geliştirmiş olduğumuz yöntem temel alınarak, (PZT) ile kantitasyonun basamakları, iç kontrolleri ve geçerlilik testleri anlatılmıştır.

**Gereç ve Yöntemler:** BCR-ABL ve ABL hedef dizileri pJET1.2 vektörlerine klonlandıktan sonra kalibratörler hazırlanmış ve gerçek zamanlı PZT tepkimelerinde kalıp olarak kullanılarak standart eğriler çizilmiştir. Bu standart eğriler kullanılarak K562 hücre dilüsyonlarının analizi yapılmıştır.

**Bulgular:** Önceden elde edilen standart eğriler kullanılarak BCR-ABL ve ABL kopya sayıları hesaplanmış; lineer sonuçlar elde edilmiştir. Tekrarlayan deneylerle yöntem duyarlılığının 1x105 hücrede bir BCR-ABL pozitif hücreyi saptayacak düzeyde olduğu gösterilmiştir.

**Sonuç:** Bu makalede tarif elden protokollerin geçerliliği/güvenirliliği gösterilmiştir. Kullanılan yaklaşım, BCR-ABL kantitasyonunun hedefleyen diğer gerçek zamanlı PZT kit ve cıhazlarına da uyarlanabilmektedir.

## INTRODUCTION

Chronic myeloid leukemia (CML) is a clonal myeloproliferativedisorder of hematopoietic stem cell origin.The Philadelphia chromosome (Ph)—der(22)t(9;22)(q34;q11)—is the hallmark of CML, and is formed viareciprocal translocation between the long arms of chromosomes9 and 22. The translocation results in the juxtapositionof the 5’ end of the BCR gene and the 3’ end ofthe ABL gene, generating a BCR-ABL chimeric oncogenewith aberrant kinase activity. The introduction of imatinibmesylate—a small-molecule competitive inhibitor of theBCR-ABL kinase—in 1998 substantially changed the managementof CML [1]. Decades of research on CML has ledto very effective molecularly targeted therapies.

Since the late 1980’s the percentage of Ph-positive cellsin bone marrow based on cytogenetic analysis has beenthe gold standard for monitoring the response to therapy[1,2,6]. Prior to the use of tyrosine kinase inhibitors forthe treatment of CML a molecular response was rarelyachieved and it was therefore not feasible to routinelymonitor BCR-ABL transcript levels, with the exceptionof patients that underwent bone marrow transplantation[1,2,3,4,5,6]. Currently, tyrosine kinase inhibitors are the preferredfirst-line therapy for CML and based on IRIS trialdata a 3-log reduction in BCR-ABL transcript levels after12 months correlates with progression-free survival [1,2,3,4,5,6].

Molecular monitoring of CML patients has becomeclinically very important. Developments in real-timequantitative (RQ)-PCR technology as well as real-timechemistry have enabled routine, high throughput detectionof BCR-ABL transcript levels. In turn, there has beenan expected increase in the number of centers that provideRQ-PCR results for BCR-ABL mRNA. The rapid increase ofthese centers revealed that there were significant variationsbetween individual laboratories in reporting RQ-PCR data[5,6,7,8,9,10,11,12]. The International Scale (IS) has been adopted forreporting BCR-ABL values based on the application of laboratory-specific conversion factors that are derived usingpatient samples [6,7,8,9,10,11,12].

Presently, few hospitals in Turkey can provide reliableRQ-PCR BCR-ABL results. Effective implementationof RQ-PCR requires knowledge of reaction kinetics andinstrumentation, as well as extensive experience. Lack ofexperienced personnel could lead to over-dependenceon the technical assistance of RQ-PCR kit manufacturers,which in turn may prove to be less than satisfactory. Hereinwe describe a cost-effective, in-house method developedin our laboratory, and detailed descriptions of validation tests and an in vitro simulation of transcript reduction,all which could be adapted to any RQ-PCR methodologychosen for detecting BCR-ABL transcript levels.

## MATERIALS AND METHODS

The study protocol was approved by the Dokuz EylülUniversity Clinic & Laboratory Reseach Ethics Committee.

**Primers and probes**

RQ-PCR results for BCR-ABL are usually presented asa ratio of the BCR-ABL transcript level and the reference control gene transcript level. Normalization to a referencegene compensates for variation in the efficiency ofthe reaction and RNA quality between samples. The mostwidely used reference control gene in BCR-ABL monitoringis the wild-type ABL gene [9,13].

**Figure 1:**
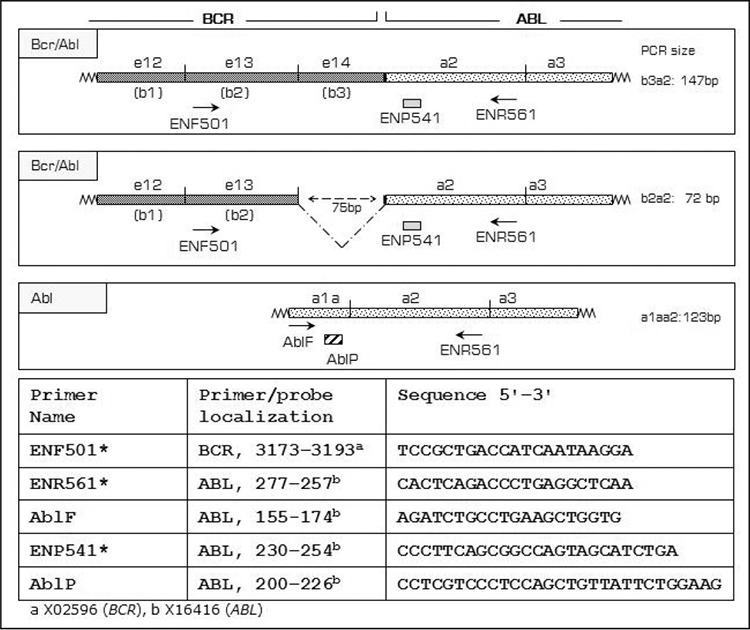
BCR-ABL fusion gene primer/probe sequences and locations (*sequences were obtained from reference 18).

PCR primers used for BCR-ABL facilitate amplificationof b2a2 and b3a2 transcripts, which comprise theBCR exon b2/b3 and ABL exon 2 (primers synthesized byMetabion GmbH, Martinsried, Germany). A dual-labeled(5’ 6-FAM and 3’ TAMRA as quencher) hydrolysis probe(TaqMan Probe, Roche Diagnostics, Mannheim, Germany)was used for real-time detection of product accumulationduring each cycle. The primer and probe sequences areshown in Figure 1. We used the wild-type ABL gene as thereference gene. The primers used most widely for quantificationof ABL also amplify the BCR-ABL fusion transcript[9,12,13]. It has been reported, and also observed by us,that this may cause a bias in results, especially when a large quantity of BCR-ABL is present in the sample [9,12,13].The primer and hydrolysis probe sequences we used forABL (Figure 1) were determined to hybridize only to wildtypeABL and not BCR-ABL.

**Cell lines**

K562 is a BCR-ABL-positive cell line derived from aCML patient in erythroid blast crisis. RS4;11 is a BCRABL-negative cell line derived from an acute myelocyticleukemia (AML) patient and was used as a BCR-ABL-negativecell line control. Both cell lines were purchased fromDSMZ (Deutsche Sammlung von Mikroorganismen undZellkulturen GmbH, Germany) and grown in RPMI-1640medium supplemented with 15% FBS and 1% L-glutaminein a 5% CO_2_-saturated incubator at 37 °C.

**Standard curves and copy numbers**

Calibrators necessary to create the standard curves forproduct quantity were generated by cloning the targetDNA into plasmids, followed by copy number determinationand preparation of serial dilutions. RNA or DNAstandards may be used for RQ-PCR, but DNA standardsare reported to have superior stability. The Europe AgainstCancer (EAC) Programme established standardized protocols for fusion transcript quantification and concluded that DNA plasmids were appropriate for constructing standard curves, whereas RNA and cDNA did not providethe stability of plasmids [10,13]. 

Total RNA was extracted from K562 cells (MachereyNagel-Nucleospin RNAII, Düren, Germany). To avoidgenomic DNA contamination RNA samples were treated with RNase-free DNase I, according to the manufacturer’sinstructions. cDNA was synthesized using a First StrandcDNA Synthesis Kit (MBI-Fermentas K1611, St. Leon-Rot, Germany), 2 μg of total RNA, and random primers,according to the manufacturer’s instructions. cDNA (5 µL)was used as the template in a conventional PCR reactionperformed with primers ENF501 and ENR561 (Figure 1)to amplify the target BCR-ABL region. The PCR productwas cloned into a pJET1.2 vector, in accordance with themanufacturer’s instructions (CloneJET PCR Cloning Kit,MBI Fermentas, St. Leon-Rot, Germany).

Following colony selection, plasmid DNA was isolated (NucleoBond^®^ PC20 Macherey Nagel, Düren, Germany) and analyzed spectrophotometrically. Similar procedureswere performed for the ABL gene. Primers AblF and ENR561 (Figure 1) were used to amplify the ABLsequence in a conventional PCR reaction, in which cDNAfrom K562 cells were used as template. The amplification product was cloned into a pJET1. 2 vector, followed by colony selection and plasmid analysis.

To prepare the calibrators the plasmid copy numberμL^–1^ volume was determined using the following equation(in brief, the molecular weight of the DNA template canbe determined by multiplying the number of base pairs(bp) by the weight of 1 mol of a bp estimated to be 650 g;using 6.022 x 1023 [Avogadro’s number] molecules mol^–1^,the number of molecules of the template can be calculatedby first converting to ng [multiplying 1 x 109] and thenmultiplying by the quantity of template) [14]

:copy number μL^–1^ = [quantity (ng μL^–1^) x (6.022 x1023)]/[plasmid length (bp) x 1.10^9^x 650] copy number μL^–1^ = [quantity (ng μL^–1^) x (6.022 x10^23^)]/[plasmid length (bp) x 1.10^9^x 650]

**Figure 2:**
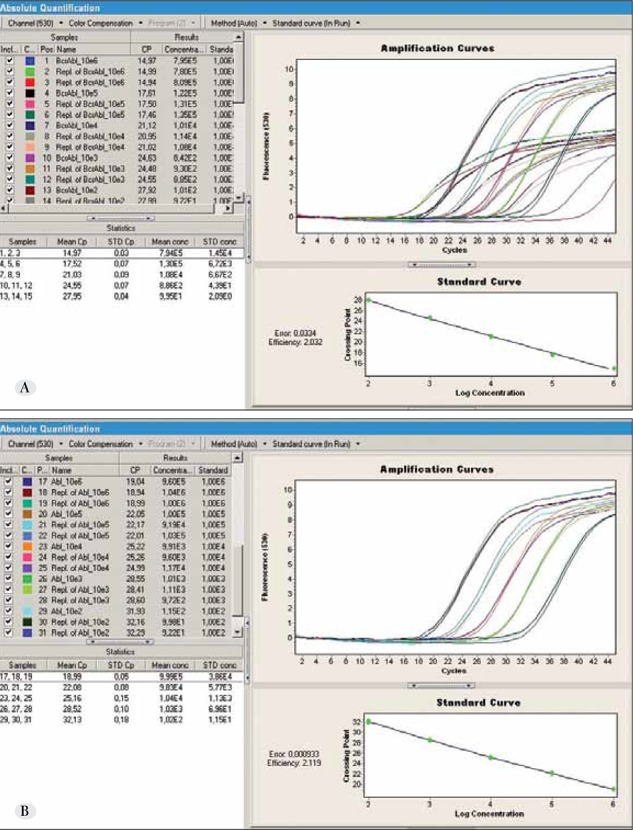
A. Standard curve, Cp values, and amplification curves for BCR-ABL during RQ-PCR. B. Standard curve, Cp values, and amplification curves for ABL during RQ-PCR

Calibrators containing 10^2^, 10^3^, 10^4^, 10^5^, and 10^6^ genecopies were prepared for both the BCR-ABL and ABL genes. These calibrators were used as templates in RQ-PCR reactionsto generate standard curves. RQ-PCR was carried outusing a LightCycler 2.0 (Roche Diagnostics, Mannheim,Germany) instrument and LightCycler TaqMan Master Kit(Roche Diagnostics, 0453528001, Mannheim, Germany).Reactions were performed in a 20-μL volume with 10pmol of each primer and probe. The same thermal profilewas optimized for BCR-ABL and ABL: pre-incubation for 10 min at 95 °C, followed by 45 amplification cycles ofdenaturation at 95 °C for 10 s, primer annealing at 58 °Cfor 40 s, and primer extension at 72 °C for 2 s. dH2O wasincluded as a no template control. Fluorescence was measuredduring the 72-°C segment in each cycle. Data wereanalyzed using LightCycler v.4.0.0.23 software (RocheDiagnostics, Mannheim, Germany). Standard curves forboth BCR-ABL and ABL were generated and saved for furtheruse (Figure 2). All samples in glass capillaries weresubsequently run in 2% agarose gel electrophoresis tocheck for size and non-specific amplifications. All datawere derived from independent experiments performed intriplicate.

**Serial dilutions of K562 cells**

Dilutions of K562 cells were prepared in the backgroundof the BCR-ABL-negative RS4;11 cell line. Mixturesof BCR-ABL-positive K562 and BCR-ABL-negative RS4;11cells were prepared, and the total cell number was always 10^5^. The sample mixtures of K562/ RS4;11 cells were preparedso that they contained 10,000, 1000, 100, 10, 1, or 0 K562 cells. These samples represent in vitro simulationof BCR-ABL transcript reduction in a CML patient undergoingtherapy. Total RNA isolation and cDNA preparationwere performed as described in the previous section.The cDNA was used as a template for determining the BCR-ABL transcript levels in quantitative PCR reactions. RQ-PCR was performed in independent triplicate sets, asdescribed in the previous section.

**Results and Interpretation**

**Figure 3:**
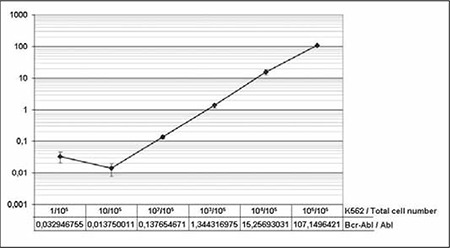
Dilutions of K562 cells were prepared in the background of the BCR-ABL-negative RS4;11 cell line and were subjected to RQ-PCR. Previously determined standard curves were used to calculate the BCR-ABL and ABL transcript copynumbers, and the results are plotted on a logarithmic scale(Y-axis). Change/reduction in the BCR-ABL transcript level is expressed as the ratio of BCR-ABL / ABL.

RQ-PCR results for the K562/RS4;11 sample mixtureswere analyzed using the previously determined standard curves. These standard curves were used to calculate thequantity of BCR-ABL and ABL transcript (copy numbers)in the samples. Change/reduction in the BCR-ABL transcript level was expressed as the ratio of BCR-ABL:ABL(Figure 3). As expected, amplification product was notobserved in the dH2O-negative controls, whereas amplifi cation was evident in the 106 copies μL–1 BCR-ABL-pos-itive control sample. From the sample mixtures of K562/RS4;11 cells only 1 sample that contained 105 RS4;11 cells(no K562 cells) was observed to be negative for the BCRABL transcript in repetitive experiments. The Cp value for ABL in this sample was very similar to other K562/RS4;11mixtures, each of which contained 105 cells, indicatingthat the result for BCR-ABL was indeed a true negative. All other sample mixtures were positive for the BCR-ABL transcript, albeit in decreasing order. Repeated experimentation showed that our methodology was able to detect 1BCR-ABL-positive cell from among 105 cells, which is thelevel of sensitivity generally accepted for BCR-ABL monitoring[15,16]. Analysis of the samples that contained varying quantities of BCR-ABL-positive cells is shown in Figure 3.

## DISCUSSION

RQ-PCR is a technically demanding, yet very powerfultool. The exquisite sensitivity of the assay is also its weakness.It is essential that extreme care be taken to avoidfalse-positive results due to cross-contamination andcarry-over of RNA/DNA from a previous amplification.The calibrators themselves are the greatest threat of contamination,as they are cloned DNA containing DNA targetregions. Their preparation, storage, and handling shouldnot be performed in the same laboratory in which patientsamples are processed. All equipment, including pipettesets, kits, reagents, paper, pens, workbooks, and lab coatsshould be dedicated for use only in that particular laboratory.It is of great importance that the rules of good laboratory practice be followed when setting up PCR assaysand during post-PCR processing of samples. Though notaddressed herein, there are excellent reviews of recommendedpre-PCR procedures in the literature, includinghandling of blood samples, RNA extraction, cDNA synthesis,and reverse transcription [8,9,10,17]. A dH2O-negativecontrol (BCR-ABL negative cells), along with low and highpositive controls should be included in each RQ-PCR runto monitor assay performance. Any changes in technique,protocol, or instrument should be accompanied by a thoroughevaluation. Standard curves should be generatedafter opening a new real-time kit or when a new batch ofprimer/probe is diluted. 

Over the past five years significant advances leadingto more wide-spread adaptation of RQ-PCR for BCR-ABLhave occurred. Implementation of the IS has improved thecomparability of results between laboratories, and recentlyaccredited reference reagents for BCR-ABL quantificationhave been developed [7]. It is essential that more diagnosticcenters in Turkey qualify to report BCR-ABL resultsaccording to the IS. In essence, the procedure involvesassignment of a laboratory-specific conversion factor (CF)to convert BCR-ABL measurements to the IS [15]. LogBCR-ABL values of the same sample set are compared toreference and local laboratories via linear regression [17].The results are considered linear when the correlationcoefficient of any 2 laboratories is >0.98 [17]. The prerequisitefor the procedure is that all in-house validationtests be performed and confirmed prior to application. Inthis context, the present study aimed to describe a costeffective,in house method for BCR-ABL quantificationand to illustrate an example for RQ-PCR validation testing,as well as to provide a description of DNA plasmidsthat may be implemented into any RQ-PCR methodologyto quantify BCR-ABL transcripts. The primary advantageof the presented methodology over widely used commercialkits—in addition to being cost effective—is that onceoptimized and validated, both absolute and relative quantificationcan be performed, whereas most commercial kitsare restricted to providing relative quantification results.In conclusion, the methodology described herein is suitablefor implementation into any RQ-PCR instrument and/or kit for quantifying BCR-ABL transcripts. 

**Conflict of Interest Statement**

The authors of this paper have no conflicts of interest,including specific financial interests, relationships, and/or affiliations relevant to the subject matter or materialsincluded.
